# The “Elephants in the Room” in U.S. global health: Indigenous nations and white settler colonialism

**DOI:** 10.1371/journal.pgph.0000719

**Published:** 2022-07-05

**Authors:** Anpotowin Jensen, Victor A. Lopez-Carmen

**Affiliations:** 1 Oglala Lakota Nation, Oceti Sakowin Land, United States of America; 2 School of Engineering, Stanford University, Stanford, California, United States of America; 3 Hunkpati Dakota Nation, Oceti Sakowin Land, United States of America; 4 Harvard Medical School, Boston, Massachusetts, United States of America; University of Calgary, CANADA

We are two Indigenous young people from the Oceti Sakowin, made up of the Lakota and Dakota Tribal Nations in modern day U.S. and Canada. As Indigenous health professionals and students, we are used to having to advocate for visibility in healthcare. In medical schools, students learn little of us and there are few to no Indigenous faculty [1]. In the academic literature, we are often left out of studies, creating a cycle of data inequity [2]. And in global health, Indigenous Nations are seldom considered, even in decolonizing global health spaces.

Earlier this year, we realized just how rooted our invisibility in global health is when we found ourselves unsuccessfully attempting to convince three white U.S. global health physicians to center and include Indigenous Peoples in a decolonizing global health paper. Instead, we were told to make a separate paper on “Indigenous issues.” When we asked the physicians to join us on the separate paper, we were told the paper would be more powerful coming from Indigenous authors alone, even after explaining the potential power of white allies joining Indigenous academics on papers that challenge the status-quo. After being siloed from the mainstream conversation on decolonizing global health, we felt this experience was a microcosm of larger systemic erasure of Indigenous Nations and white settler colonialism in U.S. global health and the decolonizing global health space.

When most U.S physicians consider the term “global health” or even the term “decolonizing global health,” they think of low- and middle-income countries (LMIC) outside their borders, usually in the global south. While countries outside U.S. borders should absolutely be included, rarely are the over 567 Indigenous Nations within U.S. borders considered, each with their own cultures, languages, spiritualities, and political systems predating colonization by thousands of years. In the field of U.S. global health and the decolonizing global health movement, Indigenous Nations and settler colonialism in the U.S. remain the elephants in the room.

From our perspective, Indigenous erasure in global health is due to the lack of recognition of our status as sovereign Nations within a high-income country that colonized us, creating massive health disparities and social determinant of health measures on par with low-and middle-income countries (LMIC) in the global south [3]. Over the past 500 years, colonization weakened Indigenous systems that helped to maintain community health (e.g. traditional food systems, access to clean water, Indigenous languages, access to land) and replaced them with unsupported and underfunded systems, leading to disproportionate systemic health disparities, including some of the highest rates of diabetes, suicide, and cardiovascular diseases [4,5]. Indigenous Peoples are exposed to pollution and pesticides, live without clean water, are less wealthy, are homeless, and experience food insecurity on levels greater than their white counterparts [6–10]. For example, the Pine Ridge Sioux Reservation, home of the Oglala Lakota Nation, has the lowest life expectancy in the U.S, and the second lowest in the entire western hemisphere [11].

Starting yesterday, U.S. global health institutions must meaningfully include Indigenous Nations within their borders as a priority. To decolonize global health, the solutions by and for Indigenous Peoples to restore their Nation’s languages, land bases, and overall well-being must be urgently supported and amplified. The problem, we have found, is that many institutions and healthcare professionals involved in global health are comfortable talking about decolonization when it comes to Nations outside of U.S. borders, but not when it calls into question violent colonization of Nations on the land they benefit from being on. This discomfort makes it difficult for non-Indigenous peoples to conceptualize healthy Indigenous Nations thriving on this land prior to the founding of the U.S., leading to a lack of understanding of the true toll of colonization on our Nations and the factors that make us healthy outside of colonial frameworks. Without the context of Indigenous Nationhood prior to colonization, the field of global health will fail to work with us in meaningful ways, just as they would for other countries that endured colonization [12].

Western institutions not seeing Indigenous Peoples as Nations is not a new phenomenon. In 1923, Chief Deskaheh of the Iroquois Confederacy, traveled from Canada to Geneva, Switzerland to attend the League of Nations (now the United Nations) to represent the Iroquois Confederacy, made up of the Mohawk, Oneida, Onondaga, Cayuga, Seneca, and Tuscarora Nations. When he arrived, the League of Nations did not let him inside the building, refusing to recognize the sovereignty of any Indigenous Nation in the Iroquois Confederacy [13]. Today, western institutions meant to promote equity among Nations, including those involved in global health, continue to see Indigenous Nations as communities, ethnic minorities, or simply a race category, rather than Nations with inherent rights to self-determination that continue to suffer from the systemic impacts of settler colonialism.

Speaking as members of the Oceti Sakowin, we invite U.S. global health to look within and work with our Nations using best practices, as they would do for countries outside their borders. We invite U.S. global health to not only see us in the context of the last 500 years, but as Nations whose journey enduring the ongoing brutality of colonization is but a small fraction of who we are. We invite U.S. global health practitioners and institutions to be brave enough to face the ongoing detrimental health impacts of colonization right here on the land beneath their feet, which means centering Native American, Alaska Native, and Native Hawaiian voices and solutions in their work. When Indigenous Peoples are not meaningfully included, U.S. institutions involved in global health are complicit in the maintenance of colonization’s violent toll on the health of our Nations. The centering of Native American, Alaska Native, and Native Hawaiian Nations in U.S. global health is not just as a moral imperative, but a practical necessity that the field must embrace to truly improve Indigenous Peoples’ health and address the ongoing detrimental health impacts of white settler colonialism on our Nations.
